# Preparation of Cu@Cu_2_O Nanocatalysts by Reduction of HKUST-1 for Oxidation Reaction of Catechol

**DOI:** 10.3390/molecules21111467

**Published:** 2016-11-02

**Authors:** Seongwan Jang, Chohye Yoon, Jae Myung Lee, Sungkyun Park, Kang Hyun Park

**Affiliations:** 1 Department of Chemistry and Chemistry Institute for Functional Materials, Pusan National University, Busan 609-765, Korea; myfilw@naver.com (S.J.); boohwallist@nate.com (C.Y.); 2 Department of Naval Architecture & Ocean Engineering, Pusan National University, Busan 609-735, Korea; jaemlee@pusan.ac.kr; 3 Department of Physics, Pusan National University, Busan 609-735, Korea

**Keywords:** nanoparticles, HKUST-1, oxidation, catechol

## Abstract

HKUST-1, a copper-based metal organic framework (MOF), has been investigated as a catalyst in various reactions. However, the HKUST-1 shows low catalytic activity in the oxidation of catechol. Therefore, we synthesized Fe_3_O_4_@HKUST-1 by layer-by layer assembly strategy and Cu@Cu_2_O by reduction of HKUST-1 for enhancement of catalytic activity. Cu@Cu_2_O nanoparticles exhibited highly effective catalytic activity in oxidation of 3,5-di-*tert*-butylcatechol. Through this method, MOF can maintain the original core-shell structure and be used in various other reactions with enhanced catalytic activity.

## 1. Introduction

Metal-organic frameworks (MOFs) have been of great interest for their well-defined porous structure and large surface area [1]. MOFs are widely applied in many fields ranging from gas sensing [2] to electronics [3] and drug delivery [4]. In addition, MOFs have been used as catalysts due to their porosity and high surface area [5]. Moreover, the high metal content in MOFs renders such materials with a highly crystalline structure. However, the low stability of some MOFs under certain reaction conditions makes them unsuitable as efficient catalysts [6]. Therefore, much research has been carried out to overcome the disadvantages of MOFs [7,8,9]. Recently, a new strategy consisting of coating MOFs with magnetic nanomaterials for enhanced catalytic activity and recyclability of the catalysts has been reported [10,11,12]. Magnetic nanomaterials exhibit superparamagnetism and they can be easily separated and recycled without serious loss of activity. Zhu et al. synthesized Au-Fe_3_O_4_@MOF nanomaterials featuring easy separation and high catalytic activity through a layer-by-layer assembly method [12]. The MOF shell thickness was easily controlled by adjusting the number of assembly cycles.

HKUST-1 (HKUST = Hong Kong University of Science and Technology), reported in 1999 by Chui et al. is a widely studied MOF in heterogeneous catalysis. The structure of HKUST-1 has a Cu unit coordinated to four BTC (1,3,5-benzenetricarboxylic acid) ligands [13]. HKUST-1 has been employed as a heterogeneous catalyst in the cyanosilylation of benzaldehyde [14], ring opening of epoxides [15] and Suzuki cross-coupling reactions [16]. In a previous report, HKUST-1 was employed as a catalyst in oxidation reactions [17]. Due to the low catalytic activity of the original MOF, the authors used a modified HKUST-1 as the catalyst for oxidation reactions. HKUST-1 showed high catalytic activity in the oxidation of phenol, but low in the oxidation of catechol.

Catechols are important substances in biology owing to their antioxidant activity [18]. However, the toxic effects of catechol, such as skin erosion, have also been demonstrated [19]. Formation of carcinoma in rodents promoted by excess catechol in rodents was reported [20]. The copper-containing metalloenzymes execute the oxidation of catechols to quinones. Therefore, a modelling study of strategies to reduce catechol using copper compound is required.

Here, we report an efficient and feasible method to prepare Fe_3_O_4_@HKUST-1 core-shell structures through a layer-by-layer assembly strategy. We synthesized a reduced form of HKUST-1 by reduction of the HKUST-1 with NaBH_4_. We characterized the reduced form of HKUST-1, which was revealed to exhibit the structure of Cu@Cu_2_O. The resulting Cu@Cu_2_O nanomaterial was successfully applied in the oxidation of catechol with high catalytic activity.

## 2. Results and Discussion

### 2.1. Catalyst Characterization

The Fe_3_O_4_ nanoparticles prepared with trisodium citrate and sodium acetate presented diameters of about 100 nm by a solvothermal method. We then synthesized Fe_3_O_4_@HKUST-1 core-shell structures through a reaction with copper acetate (Cu(OAc)_2_) and H_3_BTC after dispersing the mercaptoacetic acid (MAA)-functionalized Fe_3_O_4_ nanospheres in ethanol [21]. In addition, as-synthesized Fe_3_O_4_@HKUST-1 was calcined at a high temperature.

**Figure 1:**
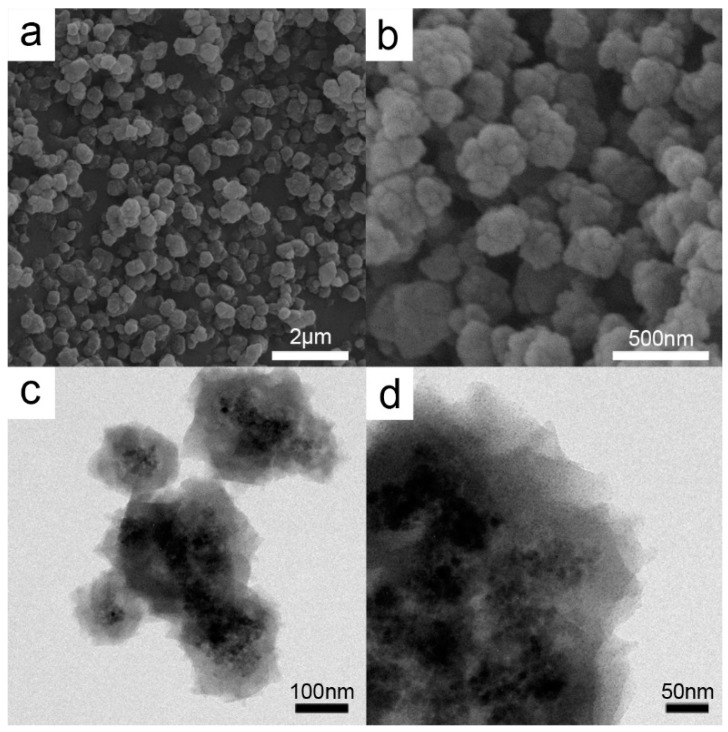
Representative SEM images (**a**,**b**) and TEM images (**c**,**d**) of Fe_3_O_4_@HKUST-1 core-shell structure.

**Figure 2:**
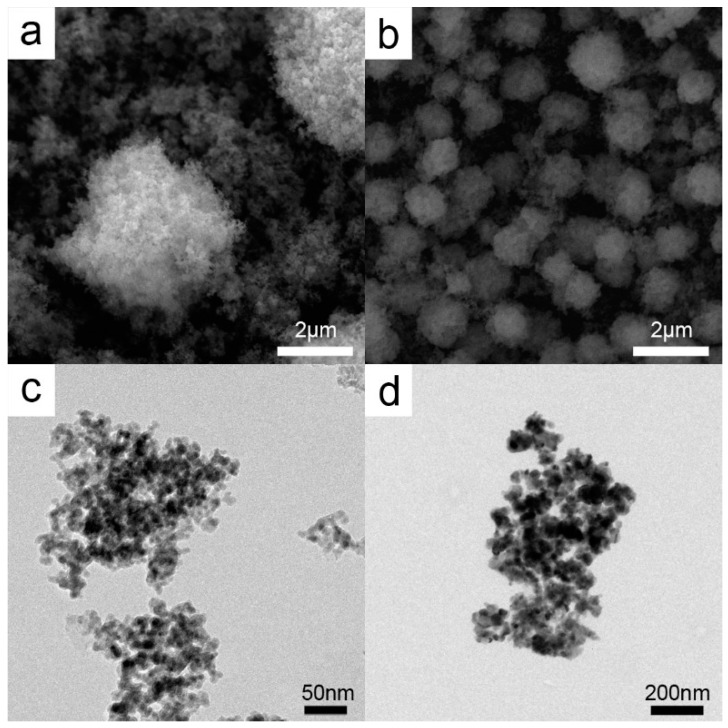
SEM images of (**a**) Cu@Cu_2_O; (**b**) calcined HKUST-1 and TEM images of (**c**) Cu@Cu_2_O; (**d**) calcined HKUST-1.

Figure 1 shows the SEM (Scanning Electron Microscopy) and TEM (Transmission Electron Microscopy) images of the prepared Fe_3_O_4_@HKUST-1. The core-shell size was less than 250 nm. The HKUST-1 samples after treatments reveal the same structure as that observed for Cu@Cu_2_O nanoparticles (Figure 2). When HKUST-1 was subjected to different treatments, the size of the particles became smaller. This observation is supported by the images of the reduced (Figure 2a,c) and calcined (Figure 2b,d) core-shell structures.

**Figure 3:**
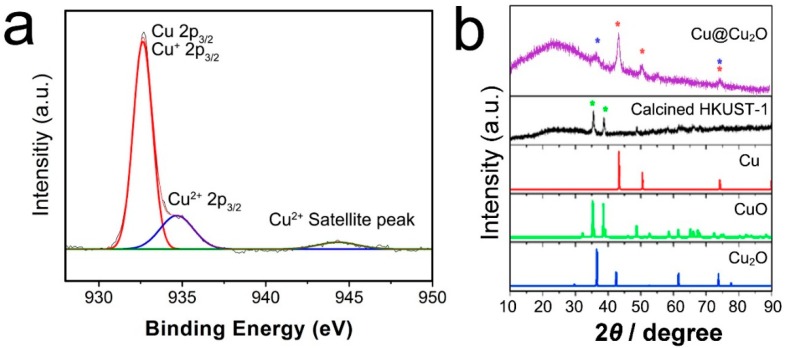
XPS Spectrum of (**a**) Cu@Cu_2_O and X-ray diffraction (XRD) patterns of (**b**) Cu@Cu_2_O and calcined HKUST-1.

The XPS (X-ray photoelectron spectroscopy) spectrum of Cu@Cu_2_O is shown in Figure 3a. As expected, Cu@Cu_2_O nanostructures showed peaks at 932.8 (Cu 2p_3/2_). Because of the small difference between binding energies of Cu and Cu_2_O (0.1–0.2 eV), determination of Cu and Cu_2_O is difficult by XPS analysis. A small amount of CuO in the nanostructure was confirmed by a small Cu^2+^ 2p_3/2_ peak (934.0 eV) and Cu^2+^ satellite peak. The powder X-ray diffraction (XRD) patterns of the two types of prepared HKUST-1 structures are shown in Figure 3b. According to the XRD patterns, the Cu@Cu_2_O sample presents an XRD pattern consistent with the data reported for Cu (JCPDS No. 04-0836) and Cu_2_O (JCPDS No. 77-0199) (Figure 3a), while the calcined HKUST-1 sample (Figure 3b) presents an XRD pattern consistent with the data reported for CuO (JCPDS No. 80-1917). We thus confirmed that the calcined HKUST-1 sample contains Cu^2+^.

### 2.2. Oxidation of 3,5-di-tert-Butylcatechol Catalyzed by HKUST-1 Structure

The HKUST-1 catalysts subjected to different treatments were employed in the oxidation of 3,5-di-*tert*-butylcatechol (Table 1). Without a catalyst, a yield of 5.5% was obtained at a temperature of 30 °C and a reaction time of 2.5 h (Table 1, entry 1). Under the same conditions, 100% yield was obtained when 5.0 mol % of the Cu@Cu_2_O catalyst was used (Table 1, entry 2). The catalytic activity of the Cu@Cu_2_O was 20 times higher than without the catalyst (Table 1, entries 1 and 2). Also, the Cu@Cu_2_O catalyst showed enhanced catalytic activity compared with HKUST-1(Table 1, entries 3, 5). This difference can probably be attributed to the changed morphology of Cu@Cu_2_O. When we used 2.5 mol % of the Cu@Cu_2_O catalyst, a 100% yield was obtained after 45 min (Table 1, entry 3). Meanwhile, calcined HKUST-1 showed lower catalytic activity than Cu@Cu_2_O in spite of increased temperature and time (Table 1, entries 3, 6). The calcined catalyst needed harsher reaction conditions in order to achieve high yields. That is, the catalyst loading, reaction temperature, and reaction time had to be increased compared to those with the Cu@Cu_2_O catalyst. The best results using calcined HKUST-1 were obtained using 5.0 mol % of the calcined catalyst at 70 °C for 3 h (Table 1, entry 8).

## 3. Materials and Methods

### 3.1. General Remarks

The morphology of the samples was analyzed by SEM on FEI Quanta 200 microscope (Thermo Fisher Scientific, Hillsboro, OR, USA) operating at 15 kV. The size and shape of the Fe_3_O_4_@HKUST-1 core-shell structures, as well as those of the reduced and calcined HKUST-1 samples, were analyzed by TEM on a JEOL JEM-2100F microscope (The JEOL Legacy, Peabody, MA, USA) at an accelerating voltage of 200 kV. The XRD patterns were recorded on a Rigaku D/MAX-RB diffractometer (Rigaku, Shibuya-Ku, Japan). The reaction products were analyzed by GC-MS on Shimadzu-QP2010 SE (Shimadzu, Kyoto, Japan).

### 3.2. Synthesis of Nanoparticles

#### 3.2.1. Synthesis of Fe_3_O_4_ Nanospheres

FeCl_3_·6H_2_O (1.4 g), trisodium citrate (0.33 g), and sodium acetate (1.9 g) were dissolved in an ethylene glycol/ethanol (36 mL/4.0 mL) mixture under stirring for 5 min. The resultant mixture was then transferred to a Teflon-lined stainless-steel autoclave (with a capacity of 40 mL) and heated at 200 °C for 10 h. The autoclave was then carefully taken out to cool to room temperature. The as-prepared products were washed with ethanol and deionized water and vacuum-dried.

#### 3.2.2. Synthesis of Fe_3_O_4_@HKUST-1 Core-Shell Structure

The as-synthesized Fe_3_O_4_ particles (0.05 g) and mercaptoacetic acid (0.29 mM, 2.67 mg) were dissolved in ethanol (10 mL) under stirring for 24 h. The resulting product was then washed with ethanol and deionized water. After that, MAA-functionalized Fe_3_O_4_ was dispersed in an ethanol (4.0 mL) solution of Cu(OAc)_2_∙H_2_O (8.0 mg) under stirring for 15 min. After washing with ethanol, the product was dispersed in an ethanol (4.0 mL) solution of H_3_BTC (10 mM, 8.4 mg) under stirring for 30 min. After washing again with ethanol, these processes were alternately repeated. Finally, the products were vacuum-dried affording a series of Fe_3_O_4_@HKUST-1 core-shell structures.

#### 3.2.3. Synthesis of Cu@Cu_2_O Core-Shell Structures

The as-synthesized HKUST-1 (0.5 g) were dissolved in deionized water (20 mL) with stirring. Sodium borohydride (10 equivalent of HKUST-1) solution was added dropwise with stirring for 10 min in an ice bath. The mixture was then subjected to stirring at room temperature for 30 min. After the reaction, the products were washed with water and ethanol, and dried under vacuum.

#### 3.2.4. Synthesis of Calcined HKUST-1 Structures

The as-synthesized HKUST-1 were calcined at 500 °C for 5 h. The temperature was increased at a rate of 2.0 °C per minute.

## 4. Conclusions

In conclusion, we synthesized Cu@Cu_2_O and calcined HKUST-1 nanostructures through reduction and calcination from HKUST-1, respectively. In addition, we designed and prepared Fe_3_O_4_@HKUST-1 core-shell structures from HKUST-1 using a versatile step-by-step assembly strategy. We applied these structures in the oxidation of catechol. Cu@Cu_2_O showed efficient catalytic activity. On the other hand, calcined HKUST-1 required harsher reaction conditions than the reduced catalyst. The MOFs maintained their original core–shell structure throughout the reduction process. These modified structures with enhanced catalytic activity could be used in other catalytic reactions.

## Figures and Tables

**Table 1:** Oxidation of 3,5-di-*tert*-butylcatechol catalyzed by prepared nanoparticles ^1^. 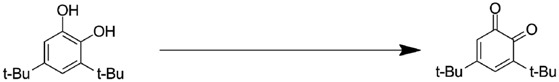

Entry	Catalyst	Temperature (°C)	Time (h)	Yield (%) ^2^
1	-	30	2.5	5
2	5.0 mol % Cu@Cu_2_O	30	2.5	100
3	2.5 mol % Cu@Cu_2_O	30	0.75	100
4	2.5 mol % Cu@Cu_2_O	30	0.5	71
5	2.5 mol % HKUST-1	30	0.75	0
6	2.5 mol % Calcined HKUST-1	50	2.5	68
7	5.0 mol % Calcined HKUST-1	60	3.0	92
8	5.0 mol % Calcined HKUST-1	70	3.0	94

^1^ Reaction condition: 3,5-DBCat 1.0 mmol, TEMPO 5.0 mol %, t-BuOK 5.0 mol %, CH_3_CN:H_2_O = 2:1, ^2^ Determined by GC/MS.

## Abbreviations

The following abbreviation is used in this manuscript:

MOF	Metal organic framework
